# Graph analysis uncovers an opposing impact of methylphenidate on connectivity patterns within default mode network sub-divisions

**DOI:** 10.1186/s12993-024-00242-1

**Published:** 2024-06-20

**Authors:** Maryana Daood, Noa Magal, Leehe Peled-Avron, Michael Nevat, Rachel Ben-Hayun, Judith Aharon-Peretz, Rachel Tomer, Roee Admon

**Affiliations:** 1 School of Psychological Sciences, University of Haifa, 199 Aba Khoushy Ave. Mount Carmel, Haifa, 31905 Israel; 2 Sakhnin College of Education, Sakhnin, Israel; 3 The Leslie and Susan Gonda Multidisciplinary Brain Research Center, Bar-Ilan University, Ramat-Gan, Israel; 4 Department of Psychology, Bar-Ilan University, Ramat-Gan, Israel; 5 Stroke and Cognition Institute, Rambam Health Care Campus, Haifa, Israel; 6 Rappaport Faculty of Medicine, Technion-Israel Institute of Technology, Haifa, Israel; 7 The Integrated Brain and Behavior Research Center (IBBRC), University of Haifa, Haifa, Israel

**Keywords:** Methylphenidate (MPH), Default mode network (DMN), Resting-state functional MRI (rs-fMRI), Functional connectivity, Impulsivity, Graph modularity analysis

## Abstract

**Background:**

The Default Mode Network (DMN) is a central neural network, with recent evidence indicating that it is composed of functionally distinct sub-networks. Methylphenidate (MPH) administration has been shown before to modulate impulsive behavior, though it is not yet clear whether these effects relate to MPH-induced changes in DMN connectivity. To address this gap, we assessed the impact of MPH administration on functional connectivity patterns within and between distinct DMN sub-networks and tested putative relations to variability in sub-scales of impulsivity.

**Methods:**

Fifty-five right-handed healthy adults underwent two resting-state functional MRI (rs-fMRI) scans, following acute administration of either MPH (20 mg) or placebo, via a randomized double-blind placebo-controlled design. Graph modularity analysis was implemented to fractionate the DMN into distinct sub-networks based on the impact of MPH (vs. placebo) on DMN connectivity patterns with other neural networks.

**Results:**

MPH administration led to an overall decreased DMN connectivity, particularly with the auditory, cinguloopercular, and somatomotor networks, and increased connectivity with the parietomedial network. Graph analysis revealed that the DMN could be fractionated into two distinct sub-networks, with one exhibiting MPH-induced increased connectivity and the other decreased connectivity. Decreased connectivity of the DMN sub-network with the cinguloopercular network following MPH administration was associated with elevated impulsivity and non-planning impulsiveness.

**Conclusion:**

Current findings highlight the intricate effects of MPH administration on DMN rs-fMRI connectivity, uncovering its opposing impact on distinct DMN sub-divisions. MPH-induced dynamics in DMN connectivity patterns with other neural networks may account for some of the effects of MPH administration on impulsive behavior.

**Supplementary Information:**

The online version contains supplementary material available at 10.1186/s12993-024-00242-1.

## Introduction

The human brain is functionally organized into distinct regions that collaboratively form extensive, interconnected neural networks [1]. Resting-state functional MRI (rs-fMRI) has emerged as a pivotal tool for delineating these large-scale networks, both in their typical function and in response to various externally induced conditions. This technique capitalizes on the observation that functionally related yet spatially separated brain regions exhibit synchronized low-frequency fluctuations in their blood oxygen level-dependent (BOLD) signals, revealing intrinsic connectivity networks (ICNs) [2]. Central to these ICNs is the Default Mode Network (DMN), that is consistently identified as a key network in rs-fMRI studies [3]. Interestingly, recent evidence suggests that the DMN is not monolithic but rather encompasses multiple intertwined sub-networks, challenging the traditional view of the DMN as a singular entity. Indeed, studies have pointed on functional heterogeneity within the DMN, proposing that the DMN can be fractionated into at least two distinct sub-networks [4–10].

The importance of studying DMN rs-fMRI connectivity patterns, as well as all other ICNs, is indicated by the notion that these networks are associated with distinct neurocognitive functions [11]. In fact, the majority of established ICNs are labeled according to their assigned neurocognitive roles, such as for example the attention, visual, auditory and somatomotor networks [12, 13]. Furthermore, connectivity patterns within and between specific ICNs were found to be differentially affected by acute pharmacological manipulations [14], as well as to predict antidepressant treatment response [15]. Abnormal connectivity patterns in specific ICNs, including the DMN, have also been linked to psychiatric conditions [16–18]. Given this background, understanding the distributed effects of acute drug administration on DMN rs-fMRI connectivity patterns becomes crucial, as it may account for the differential behavioral effects of the drug across individuals.

Methylphenidate (MPH) is an indirect dopaminergic and noradrenergic agonist and is the first line treatment for attention deficit hyperactivity disorder (ADHD) [19]. Among ADHD patients, MPH administration has been shown to modulate rs-fMRI functional connectivity patterns in the DMN [20–22], as well as in additional neural networks [21, 23–26]. Interestingly however, the few studies that directly assessed the impact of acute MPH administration on rs-fMRI connectivity patterns among healthy cohorts yielded somewhat inconclusive results [27, 28], possibly because DMN fractionation into functionally distinct sub-networks was overlooked.

One of the pathways through which MPH may improve clinical status in ADHD is via its impact on impulsivity, yielding reduced impulsive behavior [29, 30]. We recently demonstrated that a similar effect is also observed in healthy adults following acute MPH administration [31]. Notably, multiple sub-scales of impulsivity are considered to rely on DMN and dorsolateral prefrontal cortex functionality and connectivity [31–35]. In support of that, previous studies associated variability in impulsive behavior with connectivity patterns of sub-regions within the DMN network, including the medial prefrontal cortex (mPFC), posterior cingulate cortex (PCC) and anterior cingulate cortex (ACC) [36–42]. Recently, impulsivity was also linked with age-dependent alterations of functional brain networks during resting-state [43]. Taken together, these findings raise the notion that MPH-induced dynamics in DMN connectivity patterns may account for the effects of MPH administration on impulsivity.

As far as we know, the effects of MPH administration on DMN functional connectivity have yet to be defined, particularly with respect to distinct DMN sub-networks. Nor has it been explored whether these effects relate to variability in impulsivity sub-scales. In order to address this gap, fifty-five healthy young adults completed two rs-fMRI scans, following acute administration of either MPH (20 mg) or placebo, in a randomized double-blind placebo-controlled design. Analysis focused on the impact of MPH (vs. placebo) on DMN functional connectivity with all other ICNs. Graph analysis was used to fractionate the DMN into distinct sub-networks. Analyses further assessed the potential association between the impact of MPH on DMN connectivity patterns and participants’ impulsivity scores. Following previous findings, we hypothesized that acute MPH administration would yield decreased functional connectivity of the DMN with other ICNs. We further hypothesized that MPH may have a differential effect on distinct sub-networks within the DMN, and that these patterns could potentially relate to variability in impulsivity scores.

## Methods and materials

### Participants

Fifty-seven right-handed young healthy adults (32 females) were recruited to the study using online and posted ads. Inclusion criteria included age between 18 and 40, normal or corrected to normal visual acuity and right-handedness. Exclusion criteria included past or present neurological, psychiatric, or developmental disorder, head injury, substance use, chronic medication (excluding contraceptives), pregnancy or nursing and MRI contraindications. Eligible participants provided written informed consent to a protocol approved by the ethics committees of the University of Haifa (approval #368/17) and Rambam Health Care Campus (approval #0539 − 16) and received monetary compensation for their participation. Two participants were excluded due to poor MRI data quality caused by excessive head motion, yielding a final sample size of 55 participants (32 F, mean age 26.58 ± 3.91).

### Procedure

Screening procedures were employed using a combination of online questionnaires and two laboratory sessions held at the University of Haifa. The initial laboratory session involved obtaining informed consent from participants, followed by completion of the Hebrew version of the Edinburgh Handedness Inventory [44], a demographic questionnaire, and an MRI contraindication inventory. To exclude individuals with undiagnosed ADHD symptoms, participants also completed the Hebrew version of the Conners’ Adult ADHD Rating Scale [CAARS; [45]], a self-report questionnaire specifically designed to assess ADHD symptoms in adults. Consistent with established norms, participants scoring 65 or higher on the CAARS ADHD Index subscale were excluded from the study. Additionally, participants underwent clinical evaluation using the Hebrew version of the Mini-International Neuropsychiatric Interview [MINI; [46]], a brief structured interview used to diagnose Axis I psychiatric disorders. This evaluation aimed to exclude individuals with psychiatric or neurological disorders, including prior exposure to stimulant drugs or head injuries. The second session, occurring approximately 1–2 weeks later, involved the completion of the Barratt Impulsiveness Scale [BIS-11; [47]] questionnaire. Subsequently, participants were invited to take part in two neuroimaging sessions, conducted at the MRI institute of Rambam Health Care Campus. The average time interval between the two MRI sessions was 8 days (SD: 2 days). At the commencement of each session, the study’s neurologist conducted an electrocardiogram (ECG) and a pregnancy test. Afterward, participants received, via a double-blind, counterbalanced, within-subject design, a capsule containing either 20 mg of methylphenidate (MPH) or an indistinguishable placebo (PL) capsule. Heart rate and blood pressure were measured by the neurologist prior to drug administration, and ongoing monitoring of side effects was conducted throughout the sessions. Pharmacokinetic data suggest that plasma concentration of MPH reaches its peak approximately one hour after administration [48]. Accordingly, the MRI session began 45 min after drug administration to ensure peak drug blood concentration during the scan. The neuroimaging sessions involved an anatomical scan, completion of a delay discounting task [31] and a spatial attention task [49], and a resting state scan. During the resting-state scan participants were presented with a fixation cross and were instructed to lie with their eyes open.

### Measures

#### Barratt impulsiveness scale (BIS-11) questionnaire

The BIS-11 is a 30-item self-report questionnaire that assesses impulsivity across three subscales: attentional or cognitive impulsivity, non-planning impulsiveness, and motor impulsiveness [47]. Participants rate each statement, reflecting behavioral and cognitive tendencies in various situations, on a scale ranging from 1 (“never/rarely”) to 4 (“always/almost always”). Higher scores indicate a greater propensity for impulsivity. Participants completed the Hebrew version of the BIS-11 [50].

### MRI data acquisition

MRI data was acquired using a 3T GE scanner with an eight-channel high-resolution head coil, located at the MRI institute at Rambam Health Care Campus, Haifa, Israel. Functional MRI (fMRI) during the resting-state scan was carried out with a gradient echo-planar imaging (EPI) sequence of functional T2*-weighted images (TR/TE/flip angle: 2000/30/77; FOV: 240 mm; matrix size: 64 × 64) divided into 43 axial slices (voxel-size: 3mm^3^; gap: 0 mm) covering the whole cerebrum. The scan included 234 repetitions for a total duration of 7 min and 48 s. In addition, anatomical three-dimensional (3D) sequence spoiled gradient (SPGR) echo sequence was obtained at high-resolution 0.9-mm slice thickness (matrix: 256 × 256; flip angle: 12; FOV: 231 mm).

### MRI data analyses

MRI data analyses were conducted using SPM12 software (http://www.fil.ion.ucl.ac.uk/spm/software/). Data pre-processing was conducted using the CONN toolbox [version 21.A; [51]], and involved co-registration of functional and anatomical images, segmentation using SPM tissue probability maps, nonlinear volume-based spatial normalization (based on forward deformations obtained during segmentation utilizing Montreal Neurological Institute [MNI] space), and spatial smoothing with a Gaussian filter (full width at half maximum: 6 mm). The Artifact Detection Tool (ART; http://web.mit.edu/swg/software.htm) was used in order to identify and exclude outlier time points in the global mean image time series (threshold: 3 standard deviations from the mean) and movement (threshold: 0.7 mm; measured as scan-to-scan movement, separately for translation and rotation) parameters. Following pre-processing, denoising procedures included regressing out signals from the segmented CSF, white matter, the six motion parameters and their first-order derivatives, and ART volumes. Finally, data were linearly detrended and band-pass-filtered (0.008–0.09 Hz).

### Network reconstruction and analysis

To assess DMN connectivity with other ICN’s, connectivity matrices were reconstructed using the CONN toolbox. First, a whole-brain functional network was constructed for each subject, with nodes defined based on a parcellation atlas containing 300 regions of interest [11] (Figure S1). This parcellation atlas was selected because it is based on rs-fMRI data, it has a comprehensive representation of cortical and subcortical areas, and it includes a predefined set of 14 networks, including the DMN. Of this set of networks, the “unassigned” network was omitted from current analyses due to the anatomical locations of its 12 nodes, carrying high susceptibility to motion and magnetic-field artifacts. Next, edges were calculated by computing the Fisher Z-transformed Pearson correlation between the BOLD time series extracted for every two nodes, resulting in a pairwise functional connectivity matrix of 288*288 for each subject for each condition (PL and MPH). Then, a difference matrix was computed by performing paired t-test across subjects for the MPH condition vs. the PL condition for all the edges between the 65 nodes that are included in the DMN according to the atlas, and the nodes in all other 12 predefined networks. This resulted in a single matrix of 65*223. FDR correction was applied to threshold the correlation matrix and correct for the number of comparisons to control the expected proportion of falsely rejected hypotheses while maintaining higher sensitivity to detect true effects across the DMN-networks edges, yielding a Fisher Z correlation matrix of edges at *p* < 0.05 FDR corrected (range: 0.00002 < q < 0.0499). From this matrix, DMN connectivity with the other 12 predefined networks was examined by performing network-wise t-tests for all the edges per network, applying Bonferroni correction for the number of comparisons (i.e., 12 comparisons). Relations between individual differences in the impact of MPH on resting state connectivity and variability in impulsivity scores (BIS-11) were assessed using Pearson correlations.

### Graph analysis

Modular organization of neural networks was assessed using the Brain Connectivity Toolbox (BCT; available at https://sites.google.com/site/bctnet/), based on the Newman optimization method [52]. Modularity, a salient characteristic of whole-brain functional networks, refers to the extent to which a network can be partitioned into distinct, non-overlapping communities. Modularity is assessed by optimizing the configuration where intra-community connections are maximized, while inter-community connections are minimized. Within this context, a community is defined as a subset of nodes in the network (i.e., sub-network) that exhibit denser internal connectivity compared to their connectivity with external nodes in the network [52]. Here, modularity analysis was implemented to fractionate the 65 nodes of the DMN into distinct sub-networks. Fractionation was based on the impact of MPH on rs-fMRI connectivity of each DMN node with the other 12 predefined networks. Testing across levels of gamma values (γ) showed consistent results (gamma values starting from 0 and up to 10 in steps of 0.1). Hence, the resolution parameter was set to unity (γ = 1). To assess the reliability of this modular organization, the modularity detection procedure was repeated for 1000 iterations. In each iteration, the algorithm maximized the modularity Q, a metric that quantifies the strength of division of a network into modules, such that connections within modules are denser than connections between modules. Then, normalized mutual information (NMI) values were computed between each pair of the 1000 parcellations [53]. Results showed that the NMI was constantly one, indicating the same module assignment across different parcellations. Consequently, the initial partition was utilized as the modular organization.

## Results

### Impact of MPH on resting state functional connectivity of the DMN

**Fig. 1:**
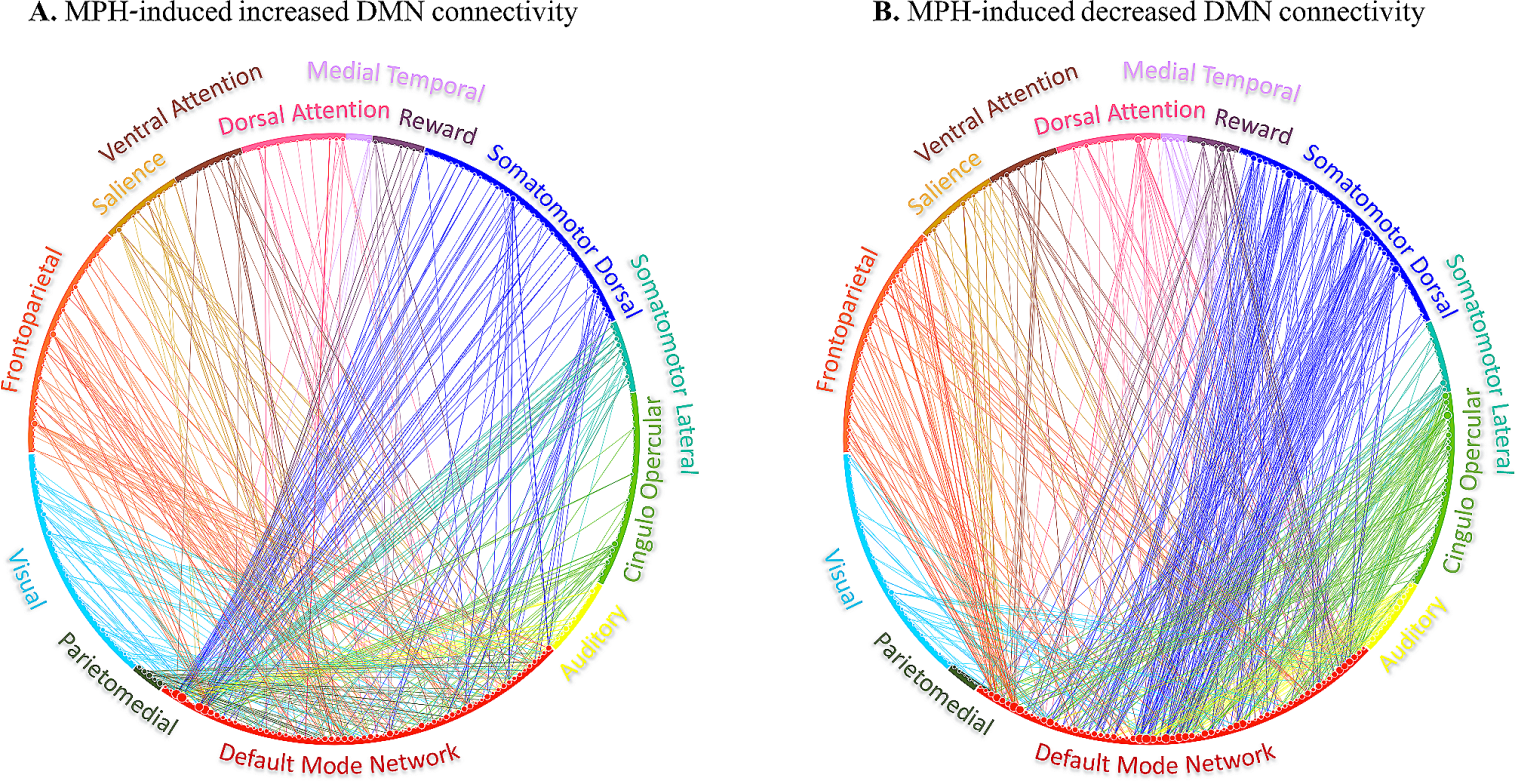
Impact of MPH on DMN resting-state functional connectivity with all other predefined networks. Nodes within the DMN with significantly increased **(A)** or decreased **(B)** functional connectivity with all other nodes following MPH compared to PL administration. All nodes are color-coded by predefined network classification. The size of the nodes, the width of the lines and their opacity depict the strength of individual connections. DMN - Default Mode Network; Methylphenidate – MPH; PL – Placebo

**Fig. 2:**
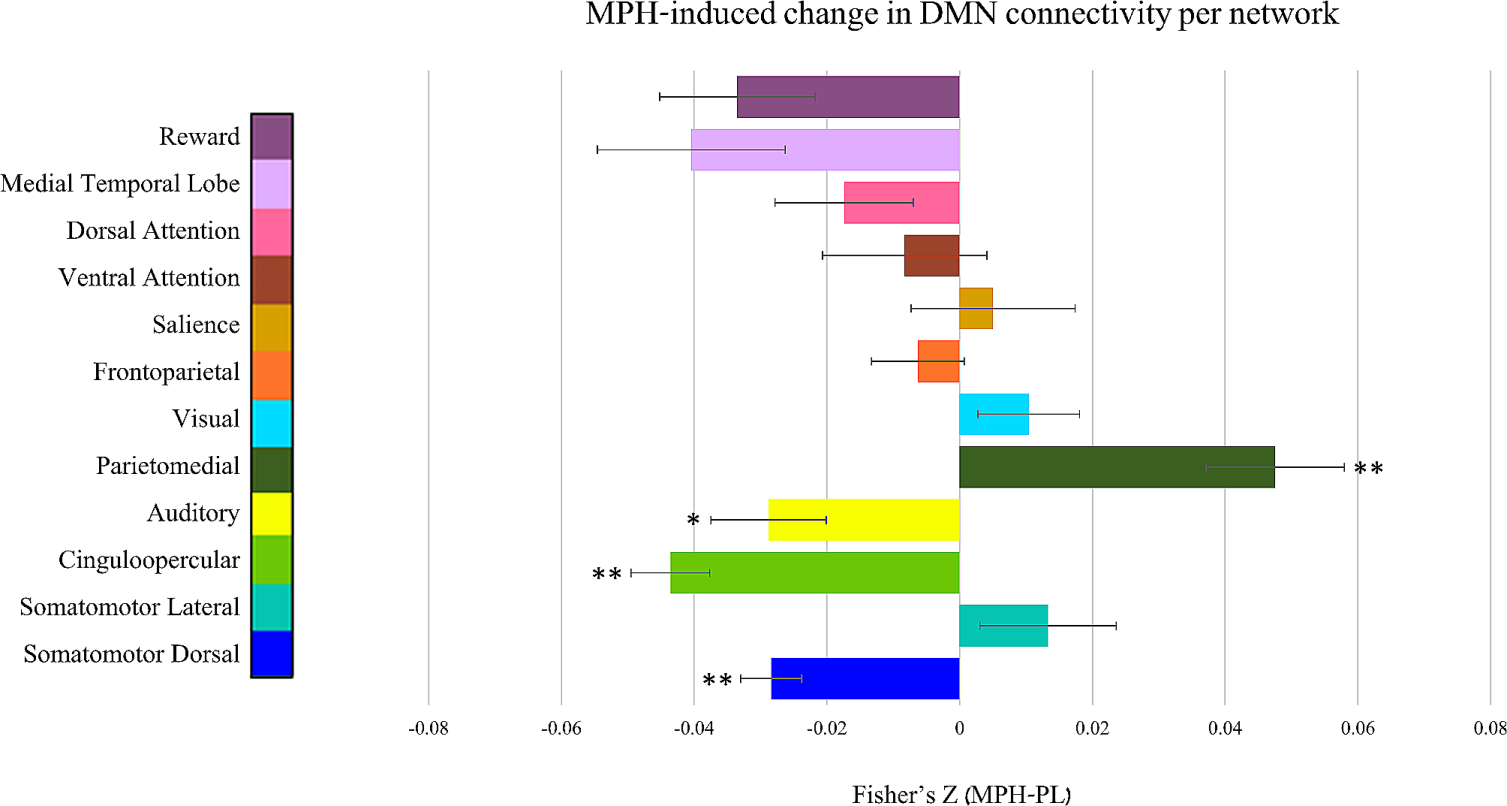
MPH-induced change in DMN connectivity per network. Impact of MPH on DMN resting-state functional connectivity with all other networks, per network (*p < 0.05, **p < 0.001; Bonferroni corrected). DMN - Default Mode Network; Methylphenidate – MPH; PL – Placebo

Paired sample t-tests for the MPH vs. PL conditions for all the edges between nodes within the DMN and all other nodes yielded a difference matrix. This matrix depicts significant changes in DMN resting state functional connectivity following MPH administration compared to placebo, corrected for the number of comparisons. Interestingly, some nodes within the DMN showed MPH-induced increased functional connectivity, while other DMN nodes showed decreased functional connectivity. These opposite effects of MPH on DMN functional connectivity patterns were found with relation to DMN connectivity with all the 12 other networks that were tested (Fig. 1). One sample t-test on all these edges revealed an overall decreased functional connectivity between nodes in the DMN and all other nodes across networks (t_[901]_ = −6.17, *p* < 0.001). One sample t-test for the connectivity of the DMN separately with nodes within each of the other 12 predefined network, corrected for the number of comparisons, revealed significantly decreased functional connectivity of the DMN following MPH administration with three specific networks (somatomotordorsal: t_[216]_ = −6.11, *p*_*Bonf. corr.*_< 0.001; cinguloopercular: t_[119]_ = −7.35, *p*_*Bonf. corr.*_< 0.001; auditory: t _[71]_ = −3.31, *p*_*Bonf. corr.*_= 0.018). In contrast, a single network increased its functional connectivity with the DMN following MPH (parietomedial: t_[38]_ = 4.56, *p*_*Bonf. corr.*_< 0.001) (Fig. 2). Supplementary Fig. 2S depicts the results of analyses assessing sex-specific differences in the impact of MPH on DMN resting state functional connectivity with all other networks.

### Impact of MPH on resting state functional connectivity of DMN sub-networks

**Fig. 3:**
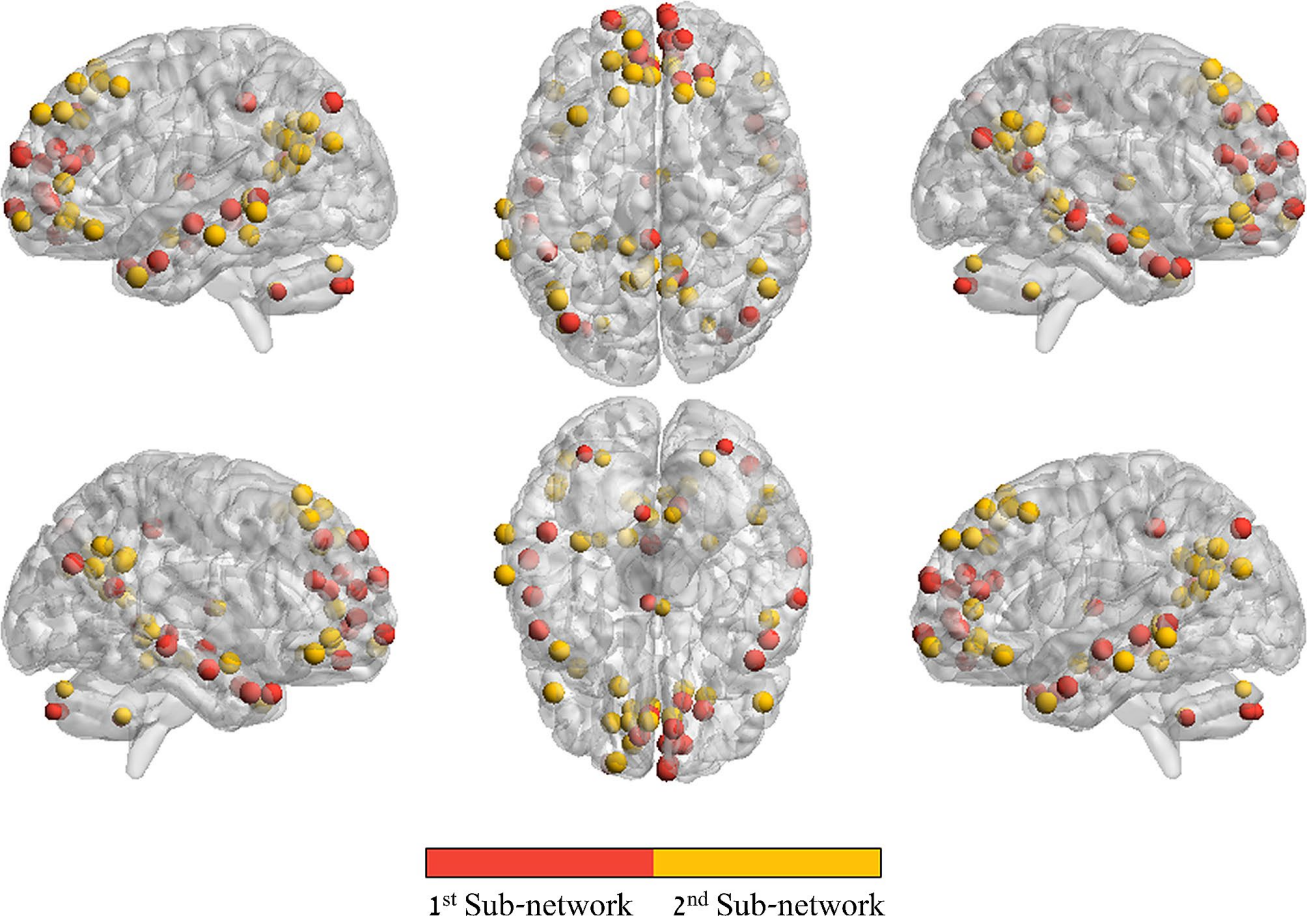
DMN sub-networks. Visualization of the two sub-networks of the DMN that emerged from the graph modularity analysis and differ in the impact of MPH on their resting-state functional connectivity. The two sub-networks share similar anatomical distribution (see Supplementary Table 1 for a specific list of coordinates)

**Fig. 4:**
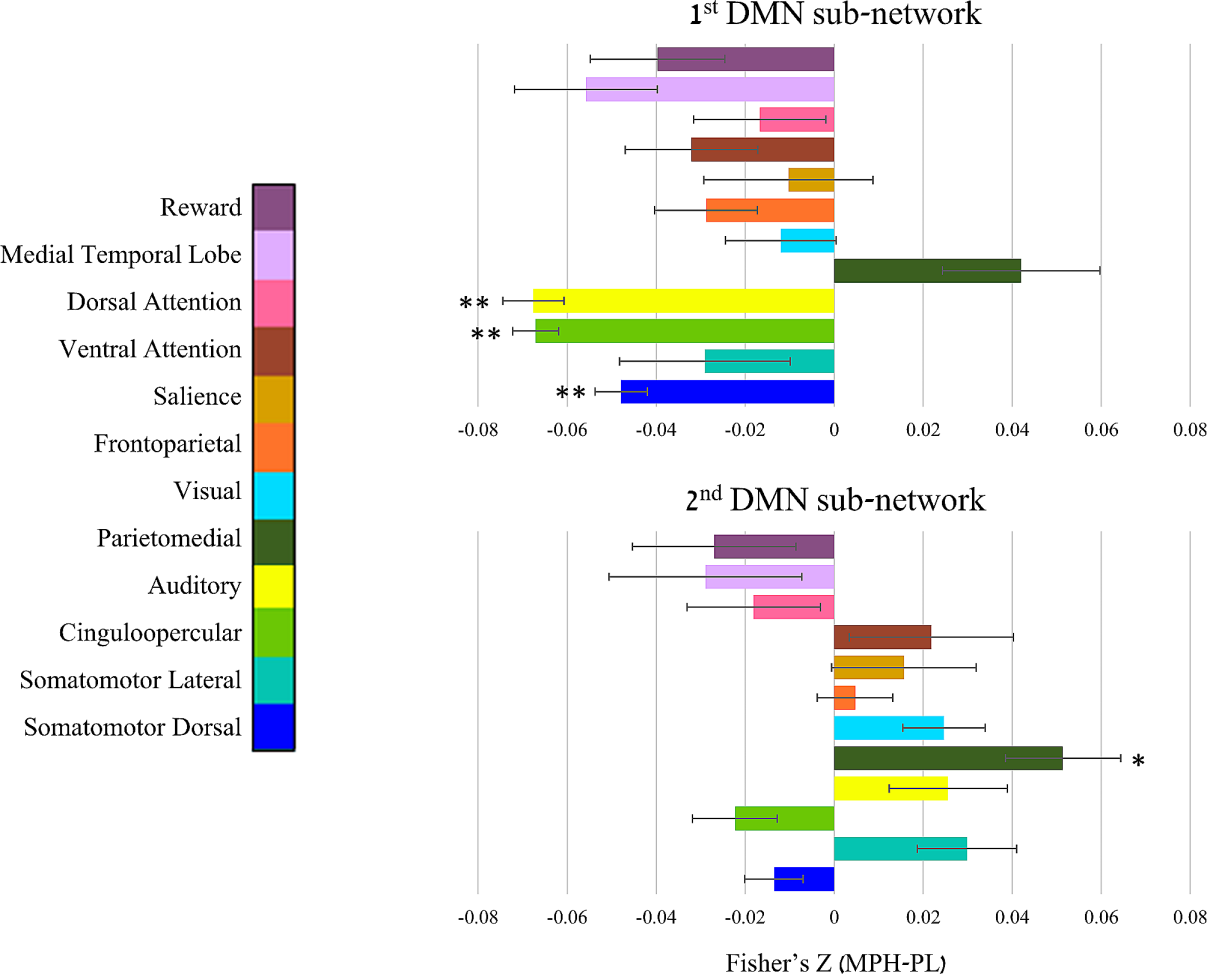
MPH-induced change in DMN sub-networks connectivity per network. Impact of MPH on DMN sub-networks resting-state functional connectivity with all other networks, per network (*p < 0.05, **p < 0.001; Bonferroni corrected). DMN - Default Mode Network; Methylphenidate – MPH; PL – Placebo

Modularity analysis revealed that the impact of MPH administration on DMN resting-state functional connectivity is best accounted for by dividing the DMN into two distinct communities of nodes (i.e., sub-networks). Of a total of 65 nodes within the DMN, the first community here included 28 nodes and the second community included 37 nodes. Interestingly, both sub-networks exhibited a similar anatomical distribution, with both sub-networks including DMN nodes in parietal, frontal and temporal lobes (Fig. 3, Supplementary Table 1). In order to uncover what drives this differentiation, one sample t-tests were performed separately for MPH-induced changes in connectivity of each DMN sub-network with all other networks, corrected for the number of comparisons. Interestingly, these analyses yielded opposite effects of MPH on functional connectivity of DMN sub-networks. For the DMN nodes included in the 1^st^ DMN sub-network, three networks decreased their functional connectivity (somatomotordorsal: t_[93]_ = −8.16, *p*_*Bonf. corr.*_ < 0.001; cinguloopercular: t_[56]_ = −12.95, *p*_*Bonf. corr.*_< 0.001; auditory: t_[41]_ = −9.84, *p*_*Bonf. corr.*_ < 0.001). For the 2^nd^ DMN sub-network, one network increased its functional connectivity (parietomedial: t_[22]_ = 3.96, *p*_*Bonf. corr.*_= 0.015) (Fig. 4).

### MPH-induced change in DMN functional connectivity and impulsivity

**Fig. 5:**
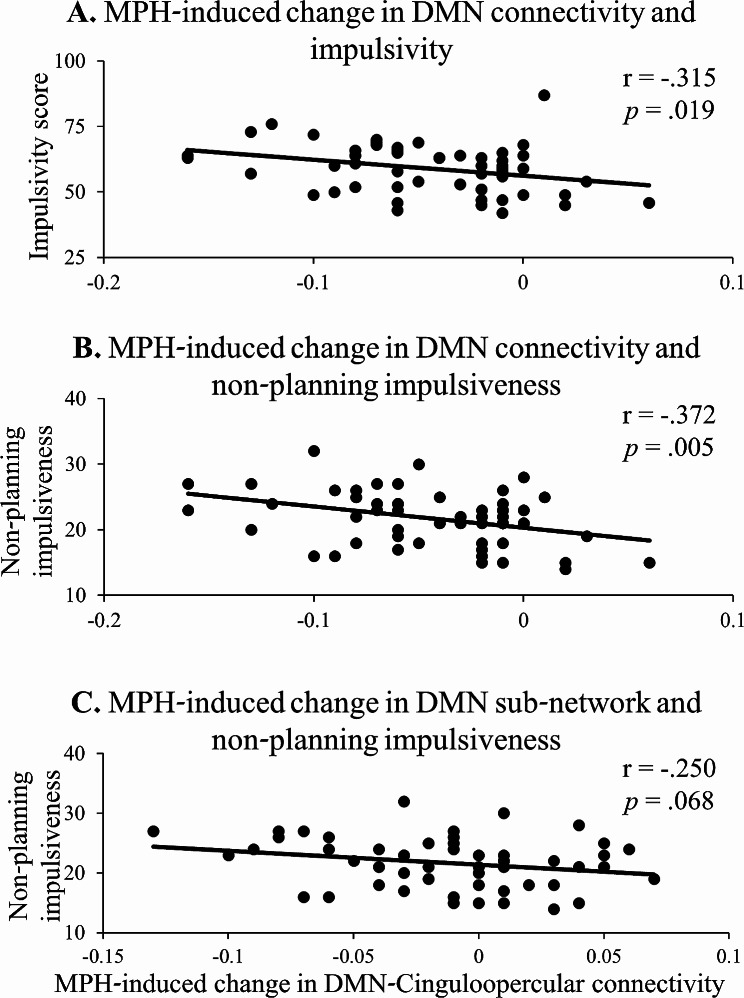
The impact of MPH on DMN and impulsivity. Negative associations between MPH-induced change in DMN resting-state functional connectivity with the cinguloopercular network and (**A**) BIS-11 total impulsivity score and (**B**) non-planning impulsiveness; as well as between MPH-induced change in resting-state functional connectivity of the first DMN sub-network, as derived from the modularity graph analysis, with the cinguloopercular network and non-planning impulsiveness (**C**). DMN - Default Mode Network; Methylphenidate - MPH

While group results revealed robust effects of MPH administration on DMN connectivity with the other predefined networks, high variability was also observed across subjects. Correlation analyses were focused on the four networks that depicted significant group effects of MPH-induced change in DMN connectivity (parietomedial, somatomotordorsal, cinguloopercular and auditory), assessing the relation between individuals’ impulsivity scores and the impact of MPH (vs. PL) on their sub-network connectivity. These analyses revealed that total impulsivity score and non-planning impulsiveness were both negatively associated with the impact of MPH on DMN functional connectivity with the cinguloopercular network (*r* = − 0.315, *p* = 0.019; *r* = − 0.371, *p* = 0.005; respectively) (Fig. 5A&B). Non-planning impulsiveness was also marginally negatively associated with MPH-induced change in resting-state functional connectivity of the cinguloopercular network with the 1^st^ DMN sub-network as derived from the modularity analysis (*r* = − 0.250, *p* = 0.068) (Fig. 5C). These results did not survive correction for multiple comparisons.

## Discussion

The aim of the current study was to examine the effects of acute MPH administration, an indirect dopaminergic and noradrenergic agonist, on resting-state functional connectivity patterns among healthy adults. We particularly focused on the connectivity of the DMN as a whole with other networks, as well as on connectivity of distinct DMN sub-networks as derived from graph modularity analysis. An additional focus was on the association between these connectivity patterns and participants’ impulsivity scores. Results revealed that, compared to placebo, MPH administration was associated with overall reduced DMN connectivity with other networks during resting-state. This is in accordance with Sripada (2013) showing decreased DMN between-network connectivity in healthy adults, and Cary (2017) showing increased segregation in multiple networks including DMN in ADHD, following MPH administration [21, 28]. Along these lines, MPH administration was also shown to increase internal DMN functional connectivity [20–22]. Considering that the DMN is highly involved in internal forms of mentalization and spontaneous cognition and is antagonistic to networks that are engaged by active attention to the external sensory environment, these findings further support the notion that MPH-induced alterations in DMN connectivity may underlie its attention-enhancing effects [54, 55]. Reduced DMN connectivity with other externally-oriented brain networks may therfore represent a neural mechanism for the cognitive enhancement properties of MPH in healthy individuals, as well as for some of its therapeutic effects in ADHD [19, 56, 57].

Importantly, while MPH decreased overall DMN connectivity, the effect was not uniform across all networks, with MPH yielding reduced DMN connectivity specifically with the auditory, cinguloopercular and somatomotor dorsal networks. Inspection of Seitzman et al., (2020) parcellation atlas reveals that nodes that are included in the cinguloopercular and somatomotor networks, as well as those in the DMN, are all part of the cortico-striato-thalamo (CST) circuit [11]. This circuit comprises the supplementary motor area, frontal eye field, lateral orbitofrontal cortex, dorsolateral prefrontal cortex, dorsal anterior cingulate cortex, putamen, caudate, globus pallidus interna, thalamus [58]. The CST circuit is posited to function as a modulatory hub exerting broad influences within and between cortical networks [7]. Previous research has demonstrated that dopamine agonists decrease, and antagonists increase, functional connectivity within this circuit [59]. Therefore, as suggested with other stimulants [26], MPH may facilitate segregation between internally oriented networks, namely DMN, and externally oriented networks, thus enhancing attention allocation towards external stimuli. In line with this suggestion, abnormal functional connectivity within the CST circuit was associated with ADHD [60], and MPH have been shown to “normalize” connectivity within the circuit [61].

MPH administration was also found to increase DMN connectivity with the parietomedial network (PMN), a relatively newly discovered neural network composed of the superior parieto-occipital fissure, posterior cingulate cortex and intraparietal sulcus. The PMN is hypothesized to be involved in memory functions and attention to internal representations [62]. Studies have demonstrate increased connectivity between the DMN and the PMN in tasks that are characterized by evaluation of internal information, suggesting that these large-scale connectivity patterns reflect temporally extended evaluation of self-generated thoughts [63]. Hence, the PMN may be seen as part of an extended DMN and its increased connectivity with the DMN following MPH in here may represent MPH-induced increases in internal DMN functional connectivity, as was demonstrated before [20–22]. This again supports the scenario that MPH may reduce the impact of DMN processing on externally oriented networks, thus increasing their potential engagement with external stimuli.

Graph analysis revealed that the DMN can be fractionated into two distinct sub-networks with divergent, even opposing, impacts of MPH administration. This is consistent with prior seed-based analyses that demonstrated MPH’s differentiated impact on DMN sub-networks both in healthy adults [64] and in ADHD [65]. Here, the first DMN sub-network was the one associated with MPH-induced reduced connectivity with the auditory, cinguloopercular and somatomotor networks, while the second DMN sub-network was associated with MPH-induced increased connectivity with the parietomedial network. These distinct component sub-networks are in accordance with previous studies showing a salient feature that includes distributed, parallel nodes within the DMN [7]. Interestingly, here, as in the majority of previous studies, DMN sub-networks did not differ with respect to their anatomical spatial distribution, such that the nodes of one sub-network lie side by side with those of the other sub-network [7, 9, 10]. Buckner and DiNicola (2019) suggested that DMN sub-networks may have originated from a less differentiated proto-organization that specialized over time [7]. Using subject-level clustering, Akiki and Abdalla (2019) suggested that the division of the DMN into two sub-networks is functionally consistent [66]. Evidence from task activation studies indicate that midline DMN structures are functionally specialized for self-relevant decisions and the inference of other people’s mental states, whereas more lateral temporal DMN components are implicated in autobiographical memory and self-oriented mental activity [67]. These findings suggest that the DMN sub-networks uncovered here may carry distinct functionality. The first DMN sub-network may tap into the CST circuit in order to suppress connectivity with external stimuli processing networks, with MPH leading to its reduced connectivity with other neural networks, hence further facilitating this segregation. The second DMN sub-network may promote attention to internal representations, with MPH increasing its connectivity with other associated networks such as the parietomedial network.

Finally, variability in the impact of MPH on DMN connectivity was associated with impulsivity scores. Specifically, MPH administration yielded a reduction in DMN functional connectivity with the cinguloopercular network, and this decreased connectivity was more potent in individuals with elevated impulsivity and non-planning impulsiveness. These results highly resemble the finding of Davis and colleagues demonstrating that differences in whole-brain functional organization are related to impulsivity [68], particularly in brain regions associated with the cinguloopercular network. Specifically, they showed decreased functional coupling between cortical control and subcortical drive modules as a function of increasing impulsivity. Previous studies have also demonstrated an association between reduced DMN- cinguloopercular resting-state functional connectivity and impulsivity, specifically relating to non-planning and disregard for future consequences [69]. In addition, the cingulo-opercular network was found to be associated with failure of response control when faced with anticipating rewards (i.e. non-planning impulsiveness) [70]. This is in accordance with our result showing that more decreased connectivity following MPH is associated with lower non-planning impulsivity. Taken together, individuals with elevated total impulsivity and specifically non-planning impulsiveness may particularly benefit from MPH administration as it leads to greater reduction in their DMN- cinguloopercular resting-state functional connectivity. Further work could examine whether distinct DMN sub-networks and intrinsic (opposed to task-induced) connectivity changes play a role in these associations.

While providing valuable insights, this study does have some limitations worth mentioning. First, we assessed the immediate impact of acute MPH administration hence cannot infer on the impact of chronic administration. Second, given that the current sample was composed of healthy adults we cannot assume that similar patterns will appear among ADHD population nor among other age groups. This is particularly relevant given the demonstrated age-dependent relation between impulsivity and functional brain networks [43], and in light of previous studies reporting on differential impact of MPH administration on resting-state connectivity patterns among healthy compared to ADHD cohorts [71]. Also important in that regard is that by relying solely on fMRI we of course cannot directly link the observed neural changes to dopamine levels. Future research employing hybrid PET/MRI techniques could provide more nuanced insights into the neurotransmitter pathways affected by MPH. It is further important to note that we administered a fixed 20 mg dose of MPH to all participants, without adjusting for individual differences in weight and/or BMI. While this dose is within the range commonly used in both clinical practice and research, future studies could benefit from exploring the effects of varying MPH doses, including weight-adjusted dosing, to fully capture variability in the effects of MPH on resting-state functional connectivity. This variability may also be influenced by plasma levels of the drug, as demonstrated by Müller et al. (2005), who found task-related fMRI signal changes to be plasma-level-dependent, emphasizing the need to consider pharmacokinetic factors in the interpretation of MPH’s neural impacts [72]. Lastly, we did not account for variability in menstrual cycle phase among female participants.

In summary, using a randomized double-blind placebo-controlled design in a sample of fifty-five healthy adults, we were able to characterize the impact of acute MPH administration on DMN resting-state functional connectivity patterns. Results revealed MPH-induced reduced DMN connectivity, particularly with the auditory, cinguloopercular, and somatomotor networks, as well as increased DMN connectivity with the parietomedial network. Graph analysis further showed that the DMN could be fractionated into two distinct sub-networks with divergent, even opposing, functionalities, such that one DMN sub-network was associated with MPH-induced reduced connectivity while the other was associated with increased connectivity. Finally, individuals with elevated impulsivity exhibited the strongest MPH-induced reduction in their DMN connectivity with the cinguloopercular network. Taken together, this study provides novel insights into the modulatory effects of MPH on DMN connectivity and its implications for impulsivity.

### Electronic supplementary material

Below is the link to the electronic supplementary material.

Supplementary Material 1

Supplementary Material 2

Supplementary Material 3

## Data Availability

Data available upon request from the authors.
