# Supplementary material for: Graph analysis uncovers an opposing impact of methylphenidate on connectivity patterns within default mode network sub-divisions
Source: Behav Brain Funct. 2024 Jun 20;20:15. doi: 10.1186/s12993-024-00242-1 (PMC11191242; doi:10.1186/s12993-024-00242-1)
Supplement: Supplementary file 1 — Supplementary Material 1 [file 12993_2024_242_MOESM1_ESM.docx]

**Figure S1**. **Functional parcellation**


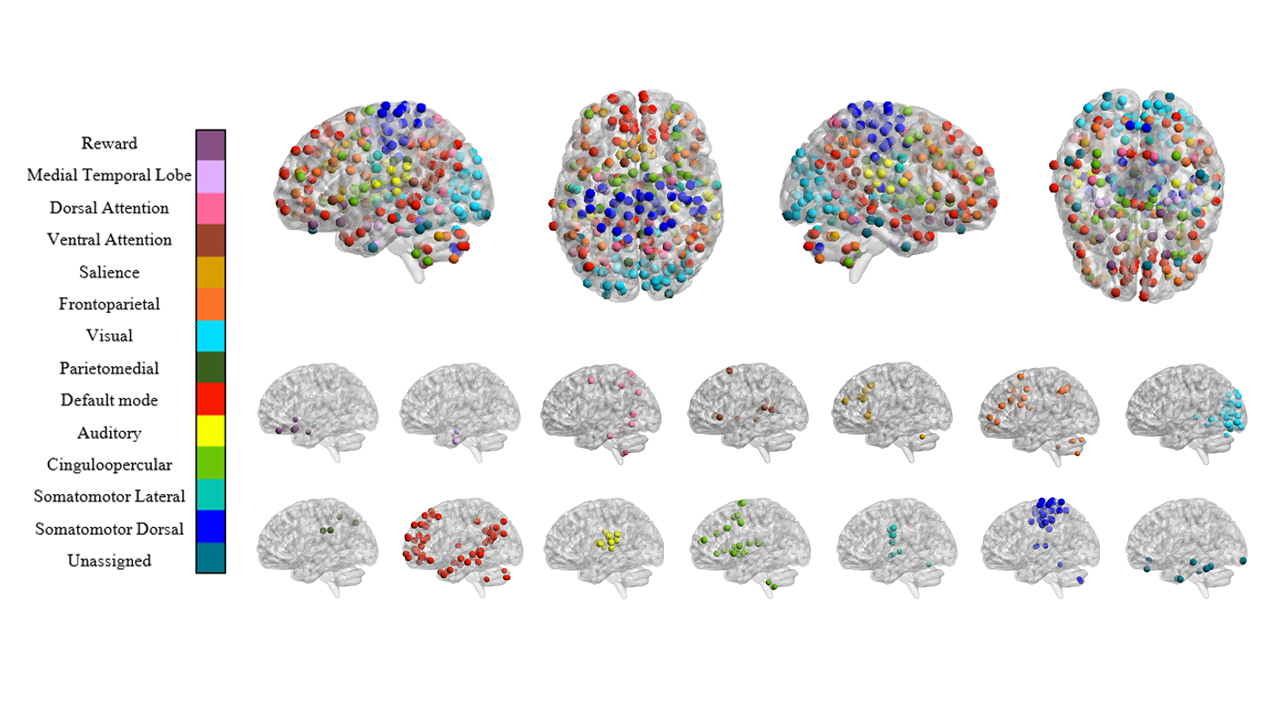


**Figure S1**. **Functional parcellation.** A cortico-subcortical functional parcellation composed of 300 nodes, subdivided into 14 predefined networks based on (Seitzman, Gratton et al. 2020). All nodes are color-coded by predefined network classification. The bottom panels include separate visual representation of each network.
