# Supplementary material for: Graph analysis uncovers an opposing impact of methylphenidate on connectivity patterns within default mode network sub-divisions
Source: Behav Brain Funct. 2024 Jun 20;20:15. doi: 10.1186/s12993-024-00242-1 (PMC11191242; doi:10.1186/s12993-024-00242-1)
Supplement: Supplementary file 2 — Supplementary Material 2 [file 12993_2024_242_MOESM2_ESM.docx]

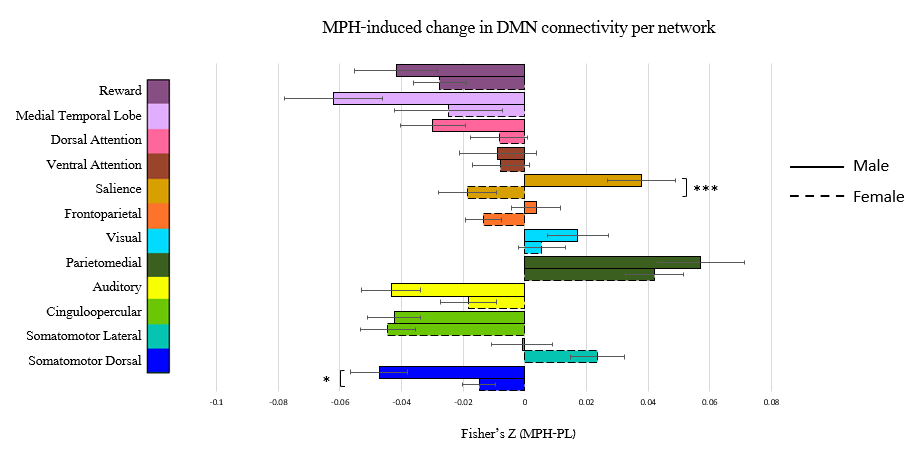
**Figure S2. Sex-specific differences in the impact of MPH on DMN resting state functional connectivity with all other networks**

**Figure S2. Sex-specific differences in the impact of MPH on DMN resting state functional connectivity with all other networks.** Comparing the strength of MPH-induced changes in DMN connectivity with all other networks between females (n=32) and males (n=23) uncovered sex-specific differences in connectivity across networks. Connectivity of the DMN with the Somatomotor Dorsal (t_[53]_ = 3.21, $p_{Bonf. corr.}$= 0.024) and Salience t_[53]_ = -3.89, $p_{Bonf. corr.}$< 0.001) networks differed between males and females. However, one sample t-test for the connectivity of the DMN with each of these two networks, separately for each sex, did not reveal significant sex-specific effects in functional connectivity following MPH administration (all p > 0.05). Therefore, we did not pursue sex-differences in the modularity analysis.
