# Supplementary material for: Graph analysis uncovers an opposing impact of methylphenidate on connectivity patterns within default mode network sub-divisions
Source: Behav Brain Funct. 2024 Jun 20;20:15. doi: 10.1186/s12993-024-00242-1 (PMC11191242; doi:10.1186/s12993-024-00242-1)
Supplement: Supplementary file 3 — Supplementary Material 3 [file 12993_2024_242_MOESM3_ESM.docx]

| **DMN Node #** | **DMN Sub-Network** | **Taliarch Daemon Lobe Atlas** | **Automated Anatomical Labeling atlas** | **x** | **y** | **z** |
| --- | --- | --- | --- | --- | --- | --- |
| 1 | 1 | Cerebellum Posterior Lobe | Cerebelum | -32 | -78 | -38 |
| 2 | 1 | Cerebellum Posterior Lobe | Cerebelum | 32 | -81 | -38 |
| 3 | 1 | Cerebellum Posterior Lobe | Cerebelum | -5.72 | -50.8 | -40.84 |
| 4 | 1 | Frontal Lobe | Frontal_Med_Orb | 5.55 | 66.69 | -3.55 |
| 5 | 1 | Frontal Lobe | Frontal_Sup | -20.16 | 63.65 | 19.39 |
| 6 | 1 | Frontal Lobe | Frontal_Sup | 12.73 | 54.87 | 38.19 |
| 7 | 1 | Frontal Lobe | Frontal_Sup | 22.11 | 39.21 | 38.9 |
| 8 | 1 | Frontal Lobe | Frontal_Sup | -7.55 | 48.08 | 23.18 |
| 9 | 1 | Frontal Lobe | Frontal_Sup | 5.94 | 54.42 | 16.18 |
| 10 | 1 | Frontal Lobe | Frontal_Sup | 6.11 | 63.98 | 21.96 |
| 11 | 1 | Frontal Lobe | Frontal_Sup | 8.8 | 54.23 | 3.45 |
| 12 | 1 | Frontal Lobe | Rectus | 8.36 | 47.59 | -15.18 |
| 13 | 1 | Limbic Lobe | Cingulum_Ant | -7.04 | 50.82 | -1.29 |
| 14 | 1 | Limbic Lobe | Cingulum_Ant | -2.5 | 41.7 | 16.05 |
| 15 | 1 | Limbic Lobe | Cingulum_Ant | 12.25 | 35.63 | 20.3 |
| 16 | 1 | Limbic Lobe | Cingulum_Mid | -2.2 | -36.68 | 43.85 |
| 17 | 1 | Limbic Lobe | Precuneus | 10.77 | -53.83 | 17.09 |
| 18 | 1 | Parietal Lobe | Parietal_Inf | -39.05 | -74.95 | 43.72 |
| 19 | 1 | Sub-lobar | Thalamus | -2.88 | -9.96 | 8.5 |
| 20 | 1 | Temporal Lobe | Occipital_Mid | 43.43 | -72.21 | 28 |
| 21 | 1 | Temporal Lobe | Temporal_Mid | -57.75 | -29.7 | -3.94 |
| 22 | 1 | Temporal Lobe | Temporal_Mid | -55.72 | -12.96 | -10.24 |
| 23 | 1 | Temporal Lobe | Temporal_Mid | -52.89 | 2.55 | -27.06 |
| 24 | 1 | Temporal Lobe | Temporal_Mid | -49.3 | -42.15 | 0.83 |
| 25 | 1 | Temporal Lobe | Temporal_Mid | 51.9 | 6.81 | -29.61 |
| 26 | 1 | Temporal Lobe | Temporal_Mid | 64.64 | -11.8 | -19.3 |
| 27 | 1 | Temporal Lobe | Temporal_Mid | 64.8 | -30.55 | -8.7 |
| 28 | 1 | Temporal Lobe | Temporal_Pole_Mid | 45.64 | 16.2 | -30.02 |
| 29 | 2 | Cerebellum Posterior Lobe | Cerebelum | -24 | -76 | -28 |
| 30 | 2 | Cerebellum Posterior Lobe | Cerebelum | 24 | -76 | -28.01 |
| 31 | 2 | Cerebellum Posterior Lobe | Cerebelum | 8 | -50 | -40 |
| 32 | 2 | Frontal Lobe | Frontal_Inf_Orb | -46.17 | 31.26 | -13.03 |
| 33 | 2 | Frontal Lobe | Frontal_Inf_Orb | 49.26 | 35.47 | -12.2 |
| 34 | 2 | Frontal Lobe | Frontal_Med_Orb | -3.06 | 44.41 | -9.46 |
| 35 | 2 | Frontal Lobe | Frontal_Mid | -35.36 | 19.86 | 50.8 |
| 36 | 2 | Frontal Lobe | Frontal_Sup | -19.78 | 45.07 | 39.48 |
| 37 | 2 | Frontal Lobe | Frontal_Sup | -16.4 | 28.52 | 53.05 |
| 38 | 2 | Frontal Lobe | Frontal_Sup | -10.33 | 54.63 | 38.71 |
| 39 | 2 | Frontal Lobe | Frontal_Sup | 23.33 | 33.07 | 47.68 |
| 40 | 2 | Frontal Lobe | Frontal_Sup | -10.09 | 39.09 | 52.29 |
| 41 | 2 | Frontal Lobe | Frontal_Sup | -2.06 | 37.85 | 36.34 |
| 42 | 2 | Frontal Lobe | Frontal_Sup | 13.08 | 29.99 | 58.65 |
| 43 | 2 | Frontal Lobe | Frontal_Sup | -17.65 | 63.19 | -9.17 |
| 44 | 2 | Limbic Lobe | Cingulum_Ant | -11.06 | 44.62 | 7.61 |
| 45 | 2 | Limbic Lobe | Cingulum_Mid | 7.94 | -48.37 | 30.57 |
| 46 | 2 | Limbic Lobe | Frontal_Med_Orb | 7.51 | 42.49 | -5.35 |
| 47 | 2 | Limbic Lobe | Precuneus | -12.6 | -39.64 | 0.93 |
| 48 | 2 | Limbic Lobe | Precuneus | -11.29 | -56.2 | 15.6 |
| 49 | 2 | Limbic Lobe | Precuneus | -6.84 | -54.9 | 27.05 |
| 50 | 2 | Limbic Lobe | Precuneus | -2.94 | -48.79 | 12.87 |
| 51 | 2 | Occipital Lobe | Precuneus | 15.12 | -63.09 | 25.98 |
| 52 | 2 | Parietal Lobe | Angular | -44.4 | -64.64 | 34.78 |
| 53 | 2 | Parietal Lobe | Angular | 46.68 | -50.08 | 28.76 |
| 54 | 2 | Parietal Lobe | Angular | 52.04 | -59.37 | 35.52 |
| 55 | 2 | Parietal Lobe | Precuneus | 5.91 | -58.82 | 35.45 |
| 56 | 2 | Sub-lobar | Thalamus | 3.42 | -7.79 | 8.23 |
| 57 | 2 | Temporal Lobe | Fusiform | -33.93 | -38.06 | -15.6 |
| 58 | 2 | Temporal Lobe | Hippocampus | -25.24 | -38.78 | -2.01 |
| 59 | 2 | Temporal Lobe | Hippocampus | 25.08 | -37.18 | -2.16 |
| 60 | 2 | Temporal Lobe | Occipital_Mid | -40.5 | -75.27 | 25.8 |
| 61 | 2 | Temporal Lobe | Temporal_Mid | -68.47 | -22.66 | -15.74 |
| 62 | 2 | Temporal Lobe | Temporal_Mid | -68.3 | -41.41 | -5.14 |
| 63 | 2 | Temporal Lobe | Temporal_Mid | -45.79 | -60.69 | 20.85 |
| 64 | 2 | Temporal Lobe | Temporal_Mid | 52.16 | -2.43 | -16.4 |
| 65 | 2 | Temporal Lobe | Temporal_Pole_Mid | -43.58 | 11.99 | -34.15 |

**Supplementary Table 1. Spatial characteristics and sub-divisions of nodes within the DMN.** Detailed overview of the 65 nodes identified within the DMN. The table depicts for each node its sub-division categorization based on current modularity analysis, its anatomical positioning according to the Talairach Daemon Lobe Atlas and the Automated Anatomical Labeling atlas, and its respective Montreal Neurological Institute (MNI) coordinates.
